# Postmortem microfocus computed tomography for noninvasive autopsies: experience in >250 human fetuses

**DOI:** 10.1016/j.ajog.2020.07.019

**Published:** 2021-01

**Authors:** Susan C. Shelmerdine, Ian C. Simcock, John Ciaran Hutchinson, Anna Guy, Michael T. Ashworth, Neil J. Sebire, Owen J. Arthurs

**Affiliations:** a Department of Clinical Radiology, Great Ormond Street Hospital for Children, London, United Kingdom; b UCL Great Ormond Street Institute of Child Health, Great Ormond Street Hospital for Children, London, United Kingdom; c Department of Paediatric Pathology, Great Ormond Street Hospital for Children, London, United Kingdom; d National Institute for Health Research Biomedical Research Centre, Great Ormond Street Hospital, London, United Kingdom

**Keywords:** autopsy, congenital anomalies, diagnosis, microfocus computed tomography, minimally invasive, miscarriage, postmortem, radiology, termination, virtual autopsy

## Abstract

**Background:**

Noninvasive imaging autopsy alternatives for fetuses weighing <500 grams are limited. Microfocus computed tomography has been reported as a viable option in small case series with the potential to avoid an invasive autopsy. Implementation of postmortem microfocus computed tomography in a large cohort as part of routine clinical service has yet been unreported, and realistic “autopsy prevention rates” are unknown.

**Objective:**

This study aimed to describe the range of abnormalities detectable on fetal microfocus computed tomography in a clinical setting and additional findings identified on the antenatal ultrasound and to estimate the invasive autopsy avoidance rate (ie, cases in which imaging was sufficient to deem autopsy unnecessary).

**Study Design:**

A prospective observational case series of all fetuses referred for microfocus computed tomography imaging at a single institution was conducted for 3 years (2016–2019). Imaging was reported by 2 pediatric radiologists before autopsy, with “decision to proceed” based on the specialist perinatal pathologists’ judgment and parental consent. Agreement rates between microfocus computed tomography and antenatal ultrasound were evaluated, and where feasible, diagnostic accuracy for microfocus computed tomography was calculated using autopsy as a reference standard.

**Results:**

A total of 268 fetuses were included (2–350 grams body weight; 11–24 weeks’ gestation), with cause for demise in 122 of 268 (45.5%). Of the 122 fetuses, 64 (52.5%) exhibited fetal anomalies. Although 221 of 268 (82.5%) fetuses had consent for invasive autopsy, only 29 of the 221 (13.1%) underwent this procedure, which implied an autopsy avoidance rate of 192 of 221 (86.9%). Complete agreement was present for all brain, thoracic, and abdominal pathologies, whereas sensitivity and specificity for cardiac anomalies were 66.7% and 91.7%, respectively. Microfocus computed tomography and antenatal ultrasound agreement was found in 219 of 266 cases (81.9%), with partial agreement in 21 of 266 (7.9%) and disagreement in 26 of 266 (10.5%), mostly because of additional cardiac, soft tissue, or genitourinary findings by microfocus computed tomography, which were not seen on the ultrasound.

**Conclusion:**

Fetal microfocus computed tomography imaging is a viable and useful tool for imaging early gestational fetuses and can avoid the need for invasive autopsy. Confirmation of antenatal diagnoses is achieved in most cases, and additional anomalies may also be detected.

**AJOG at a Glance**

**Why was the study conducted?**

This study aimed to evaluate the use of postmortem fetal microfocus computed tomography (micro-CT) as a clinical service for less invasive perinatal autopsies.

**Key findings**

Fetal anomalies were present in 25% of all micro-CT cases, and micro-CT confirmed the antenatal ultrasound findings in >80% of cases. Additional anomalies were detected on ultrasound in approximately 10% of cases. In almost 90% of cases, the perinatal pathologists did not believe additional invasive autopsy was necessary after micro-CT imaging.

**What does this add to what is known?**

Micro-CT has already been shown to have a high diagnostic accuracy rate for detecting fetal anomalies. We demonstrate that its application as a clinical imaging service is feasible, providing additional information in early gestation antenatal ultrasound alone, and could potentially avoid the need for invasive autopsies.

Current modern antenatal ultrasound techniques^1^, ^2^, ^3^ and screening tools, such as cell-free DNA,^4^^,^^5^ have led to earlier diagnoses of chromosomal and structural anomalies and earlier terminations of pregnancy (TOPs). Despite the high diagnostic accuracy of these tests, formal perinatal autopsy has traditionally remained the reference standard of care in confirming suspected anomalies, particularly to direct counseling and future pregnancy management.

However, parental acceptability of autopsy is decreasing, and perinatal autopsy at early gestational ages is technically challenging and disfiguring. Tissue breakdown after in utero demise (autolysis and/or maceration) results in a loss of anatomic detail which, combined with delicate dissection, can mean that some diagnoses are hard to determine at autopsy. Furthermore, parents are increasingly opting for noninvasive autopsy alternatives^6^ because they do not wish for large incisions.^7^^,^^8^ Larger fetuses can be imaged using magnetic resonance imaging (MRI) techniques,^9^^,^^10^ but these are nondiagnostic for fetuses weighing below 500 grams (g) body weight (approximately 18 weeks’ gestation),^11^ even at higher magnetic field strengths,^12^ because of limited image resolution.

Microfocus computed tomography (micro-CT), combined with an iodinated contrast agent, has been found to be feasible for this purpose,^13^ imaging ex vivo human fetal brains,^14^ hearts,^15^^,^^16^ and renal tissue^17^ at almost histologic resolution. Furthermore, recently published feasibility micro-CT research on imaging whole fetuses suggests high concordance rates compared with conventional autopsy.^18^ We now offer all bereaved parents the option for micro-CT imaging as part of a routine clinical autopsy service, where appropriate.

In this study, we describe the experience with this modality in terms of diagnostic yield and findings using postmortem fetal micro-CT in a large clinical dataset. Our objectives were to define the clinical diagnostic accuracy rates against an autopsy reference standard (where applicable) to describe findings detected on micro-CT compared with antenatal ultrasound and to estimate the autopsy avoidance rate through this service (ie, cases in which imaging was sufficient to deem autopsy unnecessary).

## Materials and Methods

### Patient selection

In this single center, prospective study, we reviewed all cases referred for specialist perinatal autopsy over a 3-year period (October 1, 2016 to December 1, 2019). All parents were routinely offered noninvasive imaging methods and standard autopsy as part of a perinatal autopsy investigation (including micro-CT). We included all fetuses measuring <30 cm in crown-heel length (CHL) and <5 kg (based on scanner manufacturer’s recommendations). There were no exclusion criteria.

### Tissue preparation

Before micro-CT imaging, all fetuses underwent a skeletal survey (ie, whole body radiography/babygram) and an external examination by 1 of 7 specialist pediatric pathologists (all with >10 years of pediatric pathology experience), including placental examination, where available.^19^

**Supplemental Figure 1:**
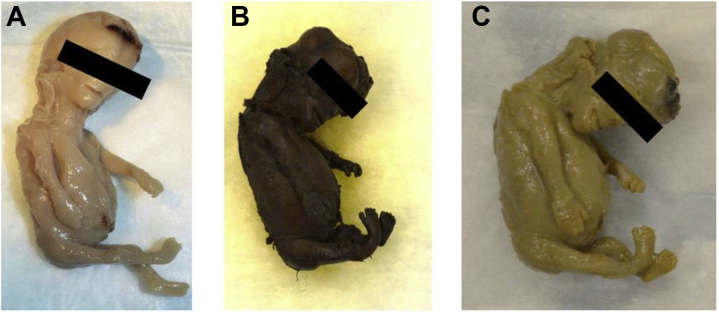
Photographs of the same 14-week-gestation fetus A, Before and B, after iodination and C, after the “deiodination” process with sodium thiosulfate pentahydrate solution. *Shelmerdine et al. Postmortem microfocus computed tomography for noninvasive autopsies. Am J Obstet Gynecol 2021*.

Fetuses were immersed in a solution of 10% formalin and potassium triiodide (I_2_KI or Lugol’s iodine; total iodine content of 63.25 mg/mL). Fetuses were stored at room temperature until fully iodinated (for ≥96 hours), rinsed, and dried before micro-CT imaging. After micro-CT, fetuses were “deiodinated” to remove superficial brown skin discoloration (Supplemental Figure 1) by immersion in sodium thiosulfate pentahydrate.^18^

### Microfocus computed tomography examination

Imaging of the fetuses were acquired using 1 of 2 micro-CT scanners located on-site (XTH 225 ST or Med-X Alpha; Nikon Metrology, Tring, United Kingdom), both equipped with a multimetal target. All imaging was undertaken by 1 of 4 trained members of the research team (S.C.S, J.C.H, A.G. or I.C.S). Fetuses were secured within the scanner using foam supports, moisture absorbent wrapping material, and Parafilm M (Bemis Company, Inc, Oshkosh, WI) to ensure mechanical stability. Imaging parameters varied according to fetal size, with X-ray energies and beam current ranging between 60 and 160 kV and 78 and 350 μA, respectively. Exposure times ranged from 88 to 1000 ms, with 1 X-ray frame per projection, with a total number of projections varying between 1066 and 3141.

Projection images acquired by the scanner were reconstructed using modified Feldkamp filtered back-projection algorithms with proprietary software (CTPro3D; Nikon Metrology, United Kingdom) and postprocessed using VGStudio MAX 3.0 (Volume Graphics GmbH, Heidelberg, Germany). Isotropic voxel sizes varied according to specimen size and magnification, ranging from 18.6 μm to 121.7 μm.

### Image analysis

All micro-CT images were evaluated and reported by 2 pediatric radiologists in consensus (O.J.A. and S.C.S., with 15 and 4 years of postmortem pediatric radiological experience, respectively). Radiologists were provided with the gestational age of the fetus, mode of death, and a brief summary of suspected prenatal fetal anomalies, if present (eg, 14 weeks’ gestation, TOP for suspected cardiac abnormality). Radiologists were blinded to the antenatal ultrasound report and images.

### Autopsy examination

The decision to perform an invasive autopsy after micro-CT imaging was guided by the imaging findings, parental consent, placental histology findings, and the pathologist’s opinion regarding potential additional benefit of further examination. For example, if the micro-CT findings were normal in the setting of an unremarkable antenatal history or confirmed the antenatal suspicions or indicated marked maceration, which would make further autopsy difficult and potentially futile, then further examination was generally not conducted. However, if there was uncertainty regarding the diagnosis on micro-CT or findings that conflicted with the antenatal examination findings, then the pathologist would take a subjective approach as to the importance of resolving this difference with an autopsy, assuming parental consent was provided, and a placental cause was not present.

All autopsies were conducted in line with the standards set by the Royal College of Pathologists.^19^ For cases in which consent was provided only for minimally invasive autopsy (MIA), these were conducted through either ultrasound-guided^20^ or laparoscopic-guided biopsy techniques.^21^ In our department, a death classification system is not used routinely, although a narrative exploration of findings linked to clinical history is provided in the final autopsy report.

### Data collection, analysis, and evaluation

Demographic details (including gestational age, mode of death [ie, termination, miscarriage], postmortem weight, crown rump length, CHL, head circumference, referred maternity unit, parental consent), time for fetal preparation (ie, days spent in iodine solution), date and results of the micro-CT imaging, antenatal ultrasound findings, and subsequent autopsy and placental histology were inputted into an independent database (Microsoft Excel, Seattle, WA).

Imaging and pathology results were all assigned as being normal, abnormal, or nondiagnostic (ie, macerated/degraded tissue) for 8 different body areas (ie, brain, spine, thorax, cardiovascular system, gastrointestinal system, genitourinary system, skeletal system, soft tissue). Free-text comments regarding the specific abnormality of each body part was also documented.

To assess the diagnostic accuracy of micro-CT, we used conventional autopsy (where performed) as the reference standard. Sensitivity, specificity, positive predictive value, negative predictive value, and concordance rates for overall diagnosis and per body part were calculated using exact methods in Microsoft Excel.

We also calculated the agreement rate in diagnoses between the most recent antenatal ultrasound report and the micro-CT findings for fetal structural anomalies. Diagnoses were compared and categorized into cases in which there was full agreement, partial agreement (ie, some pathologies detected, others not seen on ultrasound, or an anomaly identified in the same body area, but not the same type), or complete disagreement. For the purposes of calculating agreement, if there was no discordance in findings (including nondiagnostic cases by either modality), then we regarded the imaging findings to be in agreement. Descriptive statistics using percentages were used.

## Results

### Demographics

**Supplemental Figure 2:**
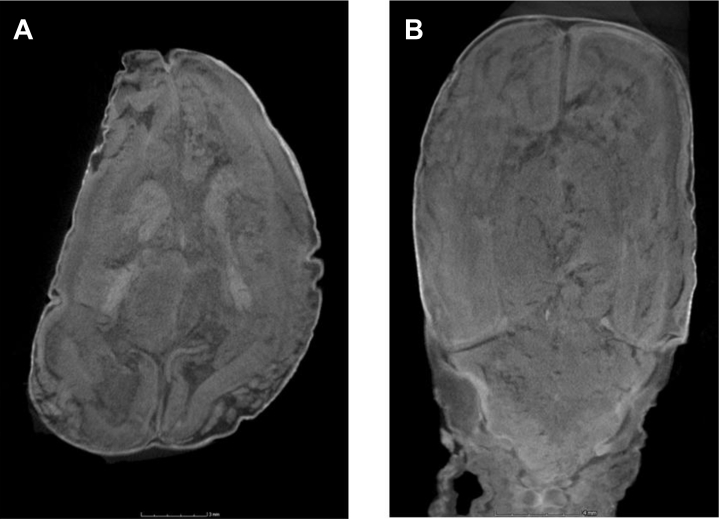
Micro-CT imaging at 35 μm resolution of a 13-week-gestation fetus with “nondiagnostic” macerated intracranial appearances A, Axial imaging at the level of the basal ganglia and B, coronal section through the posterior fossa and occipital lobes reveal extensive intracranial tissue damage, indistinct gray and white matter separation, and disrupted cerebral sulcation. *micro-CT*, microfocus computed tomography. *Shelmerdine et al. Postmortem microfocus computed tomography for noninvasive autopsies. Am J Obstet Gynecol 2021*.

Over the 38-month (approximately 3 years) study period, 268 fetuses who were referred to our institution from 11 different maternity units were imaged. The average gestational age was 16 weeks (range, 11–24 weeks), with body weight of 66 g (range, 3–350 g) and CHL of 14 cm (range, 6–26 cm). Demographic details are summarized in Supplemental Tables 1 and 2. Fetal tissue autolysis and maceration rendered several body parts noninterpretable or nondiagnostic, which included 129 of 268 brains (48.1%) (Supplemental Figure 2), 4 of 268 spines (1.5%), 2 of 268 hearts (0.8%), and 2 of 268 abdomens (0.8%).

### Major abnormalities and causes of demise

A cause of fetal demise or primary diagnosis was given in the final autopsy report in 122 of 268 cases (45.5%), with 146 of 268 (54.4%) classified as unexplained. In 12 of 146 unexplained cases (8.2%), the autopsy report stated the presence of chorionic plate or fetal membrane hemosiderosis, which was thought to be a contributing factor rather than a cause of demise, as a marker of previous hemorrhage. In 43 of 268 cases (16.0%), the placenta was not submitted for pathologic examination.

Of the cases with a definitive cause of demise or primary diagnosis, 58 of 122 (47.5%) were caused by placental pathologies. These 58 fetus deaths included 39 (67.2%) caused by chorioamnionitis, 6 (10.3%) by maternal vascular malperfusion, 5 (8.6%) by twin-twin transfusion syndrome (1 of which was caused by twin reversed arterial perfusion sequence), 4 (6.9%) by retroplacental hemorrhage, 2 (3.4%) by chronic villitis, 1 (1.7%) by parvovirus B19 infection, and 1 (1.7%) by dysmorphic placental villi favoring aneuploidy.

In 2 of 122 cases (1.6%), there was a combination of both placental and fetal abnormalities, which included 1 case of chorioamnionitis with trisomy 18 and another with chorioamnionitis in a fetus with a large cystic hygroma and complex cardiac anomalies.

The remaining 62 of 122 (50.8%) fetal deaths caused by anomalies included 15 (24.1%) with predominantly musculoskeletal and soft tissue anomalies, 6 (9.7%) with neurologic anomalies, 13 (20.9%) with multisystem anomalies, 17 (27.4%) with suspected or antenatally diagnosed genetic or trisomy abnormalities, 5 (8.1%) with cardiac anomalies, and 6 (9.7%) with abdominal abnormalities.

### Agreement between microfocus computed tomography and antenatal ultrasound

In 2 of 268 cases (0.7%), no antenatal ultrasound reports were available for review. In 1 case, the micro-CT was normal, and in the other, a cardiac anomaly (tetralogy of Fallot) was identified.

Of the remaining 266 cases for review, there was a complete agreement in imaging findings for 219 cases (81.9%). These included 188 cases (70.7%) that were normal for both antenatal ultrasound and micro-CT imaging and 31 (11.7%) in which the abnormalities on ultrasound and micro-CT were the same. Partial agreement occurred in 21 of 266 cases (7.9%), with the majority caused by multisystem (14 of 21; 66.7%) and cardiovascular anomalies (4 of 21; 19.0%).

Ultrasound and micro-CT findings disagreed in 26 of 266 cases (10.5%), which included 9 of 26 (34.6%) cases in which the micro-CT was normal but the antenatal ultrasound had identified fetal anomalies. These were mostly because of fetal hydrops (4 of 9; 44.4%) which was not evident at postmortem imaging. There was a disagreement regarding cardiac (3 of 26; 11.5%), soft tissue (3 of 26; 11.5%), and genitourinary (3 of 26; 11.5%) abnormalities in equal proportions.

The findings on ultrasound were either normal or caused by secondary signs such as raised nuchal thickness and oligohydramnios. The summary of these agreement rates by systems is presented in Supplemental Table 3, with a more comprehensive list of the individual diagnoses in Supplemental Table 4.

### Autopsy avoidance rate

Overall, 221 of 268 parents (82.5%) consented to both micro-CT imaging and invasive autopsy (eg, minimally invasive, fully invasive, or limited autopsy). After reviewing the results of the micro-CT study, pathologists proceeded with invasive investigations in 29 of 221 cases (13.1%), which resulted in an autopsy avoidance rate of 192 of 221 (86.9%).

**Supplemental Figure 3:**
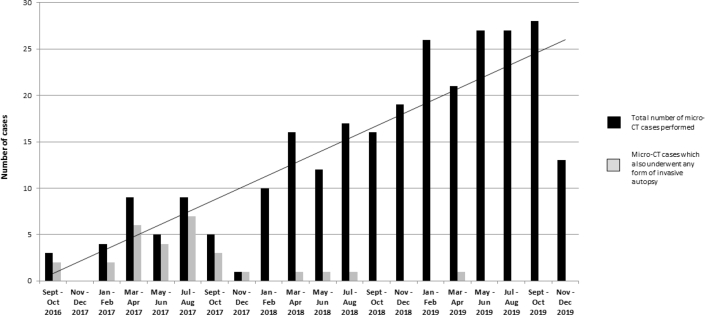
Graph depicting cases referred for postmortem micro-CT imaging over time and cases that have subsequently proceeded to any form of invasive autopsy *micro-CT*, microfocus computed tomography. *Shelmerdine et al. Postmortem microfocus computed tomography for noninvasive autopsies. Am J Obstet Gynecol 2021*.

In 47 of 268 cases (17.5%), consent was provided for micro-CT imaging and external examination of the fetus only; therefore, an autopsy avoidance rate is not applicable, but availability of micro-CT imaging allowed for a noninvasive internal review that would have otherwise not been possible, with a temporal summary of cases provided in Supplemental Figure 3. Further details on the subtype of consented autopsy and subsequent autopsy performed are detailed in Table 1.

**Table 1:** Parental preference of autopsy type (consent) and final outcome after microfocus computed tomography imaging

Parental consent	Micro-CT	MIA	Limited autopsy	Full autopsy	Invasive procedure avoided
Noninvasive autopsy (NIA) (only external, placental, and micro-CT imaging examination)	47/268 (17.5)	0	0	0	N/A
MIA (Ultrasound or laparoscopic-guided biopsies of organs+micro-CT only)	28/268 (10.4)	7/28 (25.0)	0	0	21/28 (75.0)
Limited autopsy (dissection of a predefined body part only [eg, heart]+micro-CT)	4/268 (1.5)	0	3/4 (75)^a^	0	1/4 (25)
The limited body part consented for included:					
1 for kidneys and bladders, 1 for heart only, 1 for the heart and lungs, 1 for the head and neck					
Full “invasive” autopsy+micro-CT	189/268 (70.5)	0	2/189 (1.1) (1 × presacral mass; 1 × heart and lungs only)	17/189 (8.9)	170/189 (89.9)
Total	268 (100)	7/268 (2.6)	5/268 (1.9)	17/268 (6.3)	192/221 (86.9)

Data are presented as number fraction (percentage) unless noted otherwise. The column entitled “invasive procedure avoided” was calculated by subtracting the number of cases that underwent any invasive autopsy from the total number with micro-CT imaging in each category. Denominators for calculating percentages in each row are for the total number of those who consented to each category.

*MIA*, minimally invasive autopsy; *micro-CT*, microfocus computed tomography; *N/A*, not available; *NIA*, noninvasive autopsy.

*Shelmerdine et al. Postmortem microfocus computed tomography for noninvasive autopsies. Am J Obstet Gynecol 2021*.

a Denotes the “limited autopsy” was confined to the heart (1), heart and lungs (1), and genitourinary system (1).

### Diagnostic accuracy

Invasive examinations were performed in 29 of 236 fetuses (12.3%), which included a full conventional autopsy in 17 fetuses (58.6%), an MIA in 7 (24.1%) (this subset has also been reported in previous publications^20^^,^^21^), and a limited autopsy in 5 (17.2%).

Neuropathology was performed in 12 of 29 cases (41.3%), although the brain parenchyma was too autolyzed for any definitive diagnostic conclusion in 4 of 12 cases (33.3%).

**Figure 1:**
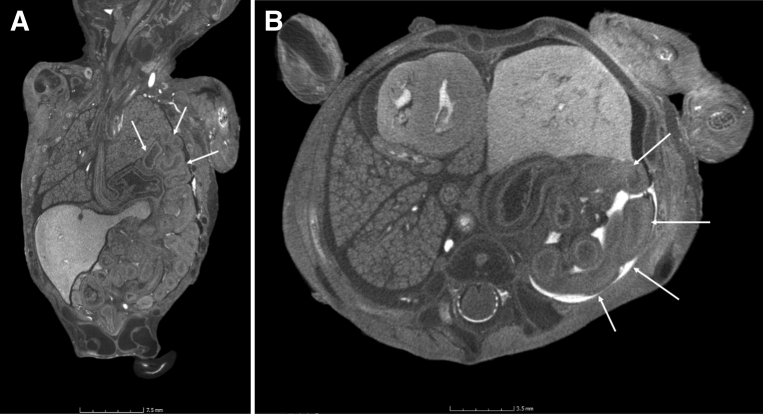
A 13-week-gestation fetus with a large left-sided diaphragmatic hernia **A**, Coronal and **B**, axial micro-CT imaging acquired at a resolution of 43 μm reveals herniation of the bowel loops, the left lobe of the liver, and the stomach within the left hemithorax (*white arrows*) with right-sided mediastinal shift of the heart. *micro-CT*, microfocus computed tomography. *Shelmerdine et al. Postmortem microfocus computed tomography for noninvasive autopsies. Am J Obstet Gynecol 2021*.

**Figure 2:**
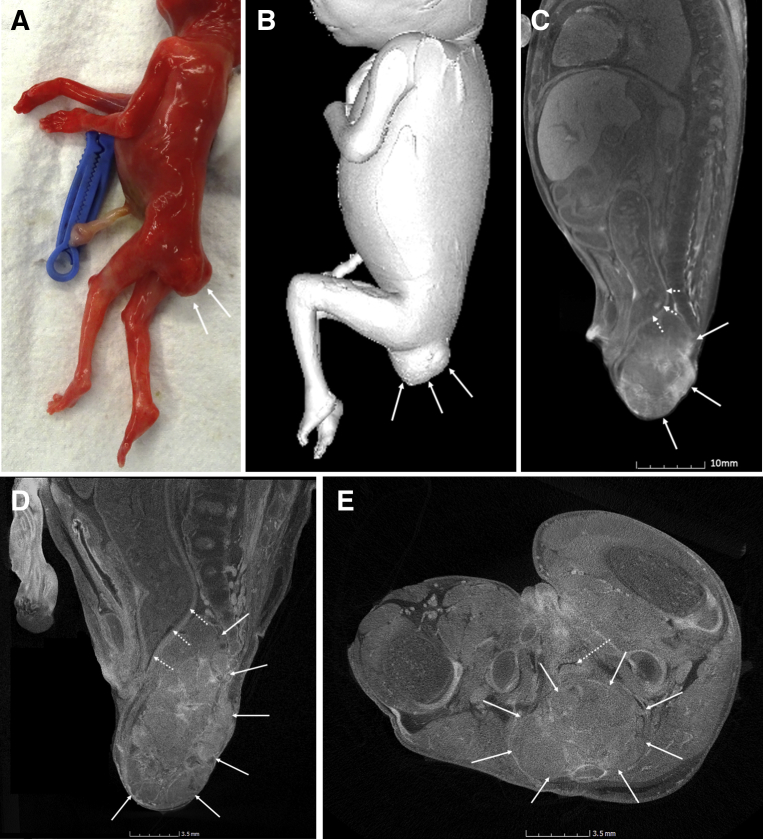
A 16-week-gestation fetus with a sacral teratoma **A**, Photograph of the fetus at external examination indicates a mass at the base of the spine (*white arrows*) and is also well shown on **B**, volume-rendered imaging of the fetus on micro-CT (*white arrows*). **C**, On the lower (76 μm) resolution views of the body, the mass is clearly visible anterior to the sacrum (*white arrows*) but posterior to the rectum (*dashed arrows*), causing proximal fecal loading and bowel distension. The higher-resolution images at 18 μm resolution of the pelvis in **D**, sagittal and **E**, axial planes reveal a heterogenous mass without any cystic spaces or focal calcification (*white arrows*). The anteriorly displaced rectum is again demonstrated by *dashed arrows*. *micro-CT*, microfocus computed tomography. *Shelmerdine et al. Postmortem microfocus computed tomography for noninvasive autopsies. Am J Obstet Gynecol 2021*.

**Figure 3:**
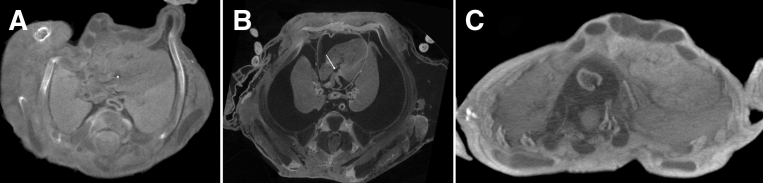
Cardiac diagnostic inaccuracies on micro-CT in different fetuses of differing gestational ages **A**, Axial micro-CT at 35 μm in a fetus at 13 weeks’ gestation in whom a ventricular septal defect (VSD) was missed (false negative) (*asterisk*). **B**, Axial micro-CT at 15 μm in a fetus at 16 weeks’ gestation with hydrops, in whom a VSD was overcalled, but was probably a capacious right ventricle (false positive) (*white arrow*). **C**, Axial micro-CT at 53 μm in a fetus at 21 weeks’ gestation in whom a VSD was overcalled (false positive). Owing to extraction-related damage and maceration, many of the false results were present in fetuses in whom normal thoracic anatomy was distorted, as clearly indicated in these 3 examples. *micro-CT*, microfocus computed tomography. *Shelmerdine et al. Postmortem microfocus computed tomography for noninvasive autopsies. Am J Obstet Gynecol 2021*.

**Supplemental Figure 4:**
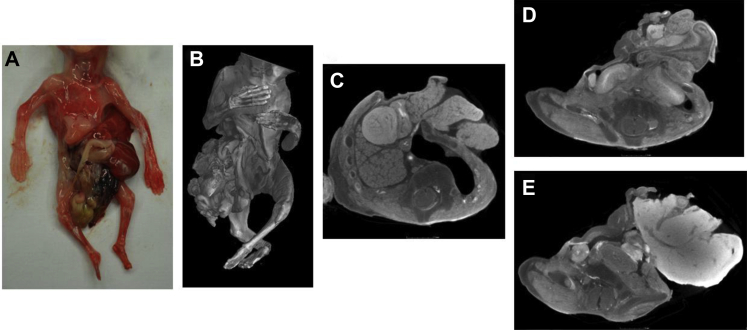
A 13-week-gestation fetus with thoracoabdominoschisis A, Photograph of the anterior thoracic and abdominal appearances at external examination. B, Volume-rendered micro-CT imaging of the fetus at micro-CT. Micro-CT imaging at 51 μm resolution, in axial planes through C, the thorax, D, the upper abdomen, and E, the lower abdomen. Although the thoracoabdominoschisis is evident on external visualization, the imaging reveals herniation of the left lung, liver, and bowel loops, whereas the adrenal glands and renal tissue remain intraabdominal. *micro-CT*, microfocus computed tomography. *Shelmerdine et al. Postmortem microfocus computed tomography for noninvasive autopsies. Am J Obstet Gynecol 2021*.

Detailed sensitivity and specificity table for each body system in this small subset is outlined in Table 2. Histology was not degraded by the iodination and sodium thiosulfate pentahydrate staining, even when some of the iodination had not been fully reversed by the process. Examples of true positives include thoracoabdominoschisis (Supplemental Figure 4), left congenital diaphragmatic hernia (Figure 1), and a presacral mass (Figure 2). Examples of false negatives include a perimembranous ventricular septal defect at autopsy which was missed on micro-CT (Figure 3).

**Table 2:** Diagnostic performance of micro-CT vs autopsy by areas of the body

Area of the body	NE autopsy	ND autopsy	ND imaging	TP/FP	FN/TN	Sensitivity (%)	Specificity (%)	PPV (%)	NPV (%)	Concordance
Nervous system	22	4	8	1/0	0/6	100 (20.7–100)	100 (61.0–100)	100 (20.7–100)	100 (61.0–100)	100 (64.6–100)
Chest	0	0	0	2/0	0/25	100 (34.2–100)	100 (86.7–100)	100 (34.2–100)	100 (86.7–100)	100 (87.5–100)
Cardiovascular system	2	0	0	2/2	1/22	66.7 (20.8–93.9)	91.7 (74.2–97.7)	50.0 (15.0–85.0)	95.7 (79.0–99.2)	88.9 (71.9–96.1)
Abdomen (non-GU)	5	1	0	3/0	0/20	100 (43.9–100)	100 (83.9–100)	100 (43.9 –100)	100 (83.9–100)	100 (85.7–100)
Abdomen (GU)	5	0	0	4/0	0/23	100 (51.0–100)	100 (85.7–100)	100 (51.0–100)	100 (85.7–100)	100 (87.5–100)
Total systems	34	5	8	12/2	1/96	92.3 (66.7–98.6)	98.0 (92.9–99.4)	85.7 (60.1–96.0)	99.0 (94.4–99.8)	97.3 (92.4–99.1)
Overall diagnosis	0	0	0	9/2	0/18	100 (70.1–100)	90.0 (69.9–97.2)	81.8 (52.3–94.9)	100 (82.4–100)	93.1 (78.0–98.1)

Nondiagnostic autopsy cases were excluded from calculations. For the 8 cases in which neuropathology examination of the brain could be performed, 7 were of diagnostic quality for comment on micro-CT imaging (in 1 case, brain imaging was too autolyzed for comment). There was 1 true positive (isolated occipital encephalocele) and 6 true negatives. A high conus medullaris position in 3 cases of caudal regression was not evaluated by pathology. The lungs were examined in 27 of 29 fetuses, of which there were 2 true positives—a thoracoabdominoschisis and a left congenital diaphragmatic hernia. There were no false positives or negatives.

The heart was examined in 27 of 29 fetuses, of which there were 2 false positives (both with suspected ventricular septal defect [VSD] on micro-CT) and 1 false negative, which was normal on imaging but found to have a perimembranous VSD at autopsy. Notably, 2 true positives were identified including 1 hypoplastic left heart syndrome (HLHS) with an atrioventricular septal defect (AVSD) and another with a hypoplastic aorta with atrial septal defect (ASD).

The genitourinary system was examined in 24 of 29 fetuses. In 1 case, micro-CT revealed a hypoplastic left kidney that was too autolyzed for pathologic examination. There were 3 true positives including 1 case of renal agenesis, 1 horseshoe kidney, and 1 case of multicystic kidneys. There were no false positives or false negatives. The remainder of the abdominal contents (excluding the genitourinary system) was examined at autopsy in 25 of 29 fetuses. There were 4 true positives (1 anorectal malformation, 1 omphalocele, 1 diaphragmatic hernia with thoracoabdominal schisis, 1 presacral mass) and no false negatives or positives.

The numbers in parenthesis under the columns for sensitivity, specificity, PPV, NPV and concordance denote 95% confidence interval values.

*FN*, false negative; *FP*, false positive; *GU*, genitourinary; *ND*, nondiagnostic; *NE*, not examined; *NPV*, negative predictive value; *PPV*, positive predictive value; *TN*, true negative; *TP*, true positive.

*Shelmerdine et al. Postmortem microfocus computed tomography for noninvasive autopsies. Am J Obstet Gynecol 2021*.

## Comment

### Principal findings

In this mixed, unselected fetal population, postmortem micro-CT imaging identified fetal structural anomalies in 25% of referred cases, and an invasive autopsy was deemed unnecessary in almost 90%. Micro-CT confirmed the antenatal ultrasound findings in >80% of cases and revealed additional anomalies in some cases.

In a small subset of cases with invasive autopsy as the reference standard, micro-CT revealed a high diagnostic accuracy rate overall, although this was slightly lower in sensitivity rates of cardiac diagnoses compared with a previously published work^18^ (66.5% vs 90.5%). This could be because of the fact that autopsy in this study was performed where the pathologist believed there could be an additional value to doing so (as opposed to the previous work where these were done in all cases) and differences in statistical calculations. In this study, we considered any cardiac abnormality that was not seen on micro-CT as inaccurate for cardiac diagnosis overall (per fetus), whereas the previous study assessed 9 cardiac indices per fetus and calculated diagnostic accuracy based on the number of inaccurate indices, rather than describing any abnormal cardiac finding as an overall inaccurate cardiac assessment. Although this method may provide more granular information on cardiac abnormalities, our method provides a more simplistic method for explaining the potential shortfalls of micro-CT in cardiac assessment to the parents.

### Clinical implications

This work represents advancement in postmortem imaging services, offering parents who have experienced an early pregnancy loss a diagnostic alternative to conventional autopsy. In the past, these cases would have been offered a binary decision of either an external examination or a full autopsy without other options, despite a recent large study finding that almost 90% of 1000 bereaved parents would prefer some form of less invasive investigation, if available.^6^ Although alternatives to micro-CT exist, such as ultrahigh-field MRI (eg, 7T and 9.4T MRI^22^, ^23^, ^24^), micro-CT is a more practical solution for a clinical service given the high cost of MRI machinery and longer scanning times. Because of the similarity in equipment and running costs between a micro-CT scanner and a medical CT scanner,^25^ micro-CT imaging offers a more affordable option for centers wishing to provide comprehensive postmortem imaging services and can be operated by healthcare professionals from different medical backgrounds after a short period (approximately 1–2 months) of dedicated supervised training. Although the staining of the fetus with iodinated solution is 1 drawback of micro-CT imaging, we did not receive any negative parental feedback.^16^^,^^26^ We have demonstrated a change in our clinical practice over 3 years as a result of regular imaging and increased confidence in the imaging results. Although the smallest fetus in this study cohort was at 11 weeks’ gestation, we have previously reported imaging feasibility to be as low as 7 weeks’ gestation,^27^ thereby widening the possible inclusion case criteria.

Although not the focus of this particular study, in some cases, micro-CT results have made a significant impact in future pregnancy counseling for parents.^28^ One example was a fetus who was at 16 weeks’ gestation, in whom ultrasound findings had included increased nuchal thickening and suspected cardiac anomaly (tricuspid atresia). The micro-CT revealed a hypoplastic aortic arch, atrioventricular septal defect, renal cortical cysts, and 4-limb polydactyly (undetected in utero), which was confirmed as Bardet-Biedl syndrome on genomic sequencing,^28^ allowing consideration of preimplantation and early gestation genetic testing in future pregnancies.

### Research perspectives

To increase the availability and applicability of micro-CT imaging for multicenter, clinical services, several issues and research avenues still remain to be addressed—these include follow-up studies to measure the actual change to clinical management in future pregnancies (assessing also whether useful additional information over the usual antenatal imaging was provided); alternative (or faster) methods for tissue staining to reduce clinical turnaround times; work surrounding parental views and expectations of the technique (such as adequacy of consenting procedures, information regarding likelihood of nondiagnostic or false results, and best methods of relaying imaging results, potentially using three-dimensional [3D] printing methods to reveal anomalies^25^); and technological advances for efficient, less costly data storage requirements (micro-CT files generate between 10 and 30 gigabyte [GB] data per case,^13^ compared with standard CT or MRI generating <1 GB per case). All of these could feed into a larger economic health costing analysis to determine the widespread feasibility of providing this service.

### Strengths and limitations

The main limitation of this study includes the observational nature of the work. This is mainly caused by a lack of additional autopsy examinations conducted (including lack of placental evaluation in some cases), thereby reducing the cohort in whom we could accurately calculate micro-CT diagnostic accuracy rates and exclude other nonstructural causes of death. It was not considered ethical to withhold the offer of a micro-CT examination to parents who refused standard autopsy (but who did request postmortem imaging) or to perform the autopsy if the pathologist did not believe there would be an added benefit. Nevertheless, this shows the potential value of micro-CT examination to offer a service for parents who refuse standard autopsy and would traditionally otherwise receive no fetal investigation. In addition, because this was a clinical service, reporting pathologists were aware of the micro-CT imaging findings before any subsequent invasive autopsy, hence directing the investigation. Although the small number of cases and nonblinded fashion of reporting limit an objective measure of diagnostic accuracy, they do provide a realistic reflection of this approach in real-world clinical practice. High-resolution 3D imaging has changed clinical fetal postmortem examination practice, increasing confidence in pathologists to not proceed to formal autopsy in these challenging cases.

Furthermore, some areas of apparent disagreement between micro-CT and antenatal ultrasound findings were unable to be resolved in our study, because formal autopsy was not performed. Although the high resolution and magnification of our imaging technique are superior to first- and second-trimester fetal ultrasound imaging^2^^,^^29^^,^^30^ and may be superior to standard autopsy as reported in a previous blinded study,^18^ we were unable to formally confirm the presence or absence of some of the abnormalities reported.

Finally, micro-CT imaging appropriateness and usefulness may not be generalizable to different populations. It is possible that mothers who have experienced recurrent miscarriages without medical explanation or TOPs or who had abnormal antenatal imaging results may have been more likely to consent to our study. In addition, those from religious or ethnic minority groups may have been more willing to consent to noninvasive imaging in preference to autopsy irrespective of referral criteria.^31^ Other centers’ population demographics may make micro-CT imaging at these gestations even more desirable.

### Conclusion

Our data indicate that postmortem micro-CT imaging for human fetuses as part of a noninvasive autopsy examination is a feasible and growing service, with high diagnostic accuracy rates in a small subset in which autopsy was performed. It can provide additional information over the antenatal imaging results and has resulted in a reduction in subsequent invasive autopsies being performed locally for early gestation fetuses. Further research is required regarding large-scale accuracy studies across different demographic groups and to evaluate potential economic healthcare benefits at a national level.

## Figures and Tables

**Supplemental Table 1:** Case demographics for continuous variables measured (n=268)

	Average	Median	Mode	Range
Gestational age (wk)	16	16	16.0	11–24
Postmortem weight (g)	66	42	25.0	3–350
Head circumference (cm)	10	9	11.0	4–17
Crown-heel length (cm)	14	14	20.5	6–26
Crown rump length (cm)	10	10	9.0	4–19
Time between delivery and immersion in iodine (d)	8	8	6.0	2–22
Time spent in iodine solution (d)	5	5	5.0	1–17
Total time from delivery to imaging (d)	14	13	12.0	0–48

*Shelmerdine et al. Postmortem microfocus computed tomography for noninvasive autopsies. Am J Obstet Gynecol 2021*.

**Supplemental Table 2:** Demographic information for the categorical data variables measured in our study cohort

	Total number	Percentage (n=268)
Sex		
Male	129	48.1
Female	122	45.5
Indeterminate	17	6.3
Mode of death or demise		
Termination of pregnancy	71	26.5
Miscarriage	163	60.8
Intrauterine fetal death	34	12.7
Maceration score		
0—none	82	30.6
1—mild	34	12.7
2—moderate	14	5.2
3—marked or extensive	138	51.5

*Shelmerdine et al. Postmortem microfocus computed tomography for noninvasive autopsies. Am J Obstet Gynecol 2021*.

**Supplemental Table 3:** Agreement between micro-CT imaging and antenatal ultrasound for fetal structural anomalies

Micro-CT findings	Antenatal ultrasound results		
	Agree	Partially agree	Disagree
Single-system findings			
Brain anomalies	1	1	1
Spine anomalies	0	0	2
Cardiovascular anomalies	4	4	3
Abdominal anomalies	3	0	1
Genitourinary anomalies	1	0	3
Soft tissue anomalies	8	2	3
Musculoskeletal anomalies	5	0	2
Multisystem anomalies	9	14	2
Normal micro-CT examination	188/266 (70.7%)	0	9/266 (3.8%)
Total	219/266 (81.9%)	21/266 (7.5%)	26/266 (10.5%)

The full individual list of disorders within each category is presented in Supplemental Table 4. In addition to the abovementioned findings, 2 women did not attend for any antenatal examinations; therefore, n=266.

*micro-CT*, microfocus computed tomography.

*Shelmerdine et al. Postmortem microfocus computed tomography for noninvasive autopsies. Am J Obstet Gynecol 2021*.

**Supplemental Table 4:** Disagreement between micro-CT findings and antenatal ultrasound results

Micro-CT results	Antenatal ultrasound results		
	Agree	Partially agree	Disagree
Brain anomalies			
Absent corpus callosum, interhemispheric cyst	1		1, normal
Occipital encephalocele		1, occipital encephalocele+echogenic kidneys	
Spine anomalies			
Neural tube defect			1, partial mole
Spinal cord terminating at T6			1, ventriculomegaly
Cardiovascular anomalies			
VSD	1		1, oligohydramnios
AVSD	2	1, univentricular heart	
HLHS, VSD	1		
Tetralogy of Fallot			1, raised NT
Bilateral SVCs			1, normal
DORV, ASD		1, HLHS, AVSD	
HLHS, aortic hypoplasia		1, HLHS	
Interrupted aortic arch, VSD		1, DORV, TGA, VSD	
Abdominal anomalies			
Omphalocele	2		1, normal
Sacrococcygeal teratoma	1		
Genitourinary anomalies			
Bilateral cystic kidneys	1		
Ambiguous genitalia			1, normal
Right adrenal cyst			1, normal
Horseshoe kidney			1, VM, kidneys not visualized
Soft tissue anomalies			
Amniotic band syndrome	1		1, normal
Hydropic	2	1, raised NT 1, hydropic+echogenic kidneys	1, normal
Arthrogryposis	4		
Amniotic band syndrome	1		
Decompressed cystic hygroma			1, normal
Musculoskeletal anomalies			
Four-limb phocomelia, exomphalos, cleft palate	1		
Thanatophoric dysplasia	1		
Ectrodactyly of limbs	1		
Osteogenesis imperfecta	1		
Fibular dimelia	1		
Achondrogenesis			1, hydrops and suspected cardiac anomaly
Chondrodysplasia punctata			1, normal
Multisystem anomalies			
Anencephaly, ectopia cordis	1		
Craniorachischisis, double inlet left ventricle, omphalocele	1		
Large AVSD, hepatomegaly, intracranial ventriculomegaly in the setting of trisomy 21	1		
Gastroschisis and tetralogy of Fallot	1		
Asymmetric FGR	1		
Caudal regression—single lower limb, renal anomalies	1		
Body stalk anomaly	1		
Right PFFD, megaphallus, anal atresia, small kidney, cloaca	1		
Encephalocele, cystic kidneys, polydactyly (Meckel-Gruber syndrome)	1		
NTD+renal anomalies			1, raised NT 1, IUD at initial US already
Microcephalic, macerated fetus with right-arm anomaly limbs.		1, omphalocele, cystic hygroma, microcephaly, right arm anomaly	
Micromelic skeletal dysplasia+cystic kidneys		1, thanatophoric dysplasia	
VACTERL—short tibia and fibula, polydactyly of feed, hemivertebrae, renal agenesis, VSD, no aortic arch		1, hemivertebrae and cardiac anomalies detected only	
VACTERL spectrum—vertebral anomalies, spinal dysraphism, left SVC, bilateral renal aplasia, absent bladder, anorectal malformation		1, anhydramnios; could not visualize bladder and kidneys	
Short ribs, hemivertebrae, cystic hygroma, renal cysts, AVSD, small pulmonary arteries		1, cystic hygroma, dilated renal pelvis, and RVOT	
NTD with frontal polymicrogyria		1, NTD	
VM, cystic hygroma, tetralogy of Fallot, crossed fused ectopia of kidneys		1, cystic hygroma, renal fusion, hemivertebrae, posterior fossa anomalies	
Body stalk anomaly and NTD		1, body stalk anomaly	
Micrognathia, cleft palate, dextrocardia, intestinal malrotation		1, micrognathia, cleft palate, suspected cardiac anomaly	
Caudal regression—gastroschisis, absent sacrum		1, gastroschisis; spine and limbs not visualized	
Bardet-Biedl syndrome—cystic kidneys, hypoplastic arch, AVSD, polydactyly		1, increased NT, tricuspid atresia	
Abdominal wall defect, horseshoe kidney, normal heart		1, exomphalos; suspected complex cardiac anomalies	
Caudal regression—diaphragmatic hernia, single kidney, limb anomaly		1, diaphragmatic hernia, limb anomaly, and possible cardiac defect	
Caudal regression—renal agenesis, anorectal malformation, absent sacrum and left lower limb		1, dysplastic kidneys and left leg anomaly	
Normal micro-CT examination			
	188		4, hydrops
			1, cleft palate, hydrops, major heart defect
			1, VM, small cerebellum
			1, cystic hygroma
			1, cystic structure adjacent to urinary bladder
			1, micrognathia

*ASD*, atrial septal defect; *AVSD*, atrioventricular septal defect; *DORV*, double outlet right ventricle; *FGR*, fetal growth restriction; *HLHS*, hypoplastic left heart syndrome; *IUD*, intrauterine device; *micro-CT*, microfocus computed tomography; *NT*, nuchal thickness; *NTD*, neural tube defect; *PFFD*, proximal focal femoral deficiency; *RVOT*, right ventricular outflow tract obstruction; *SVC*, superior vena cava; *TGA*, transposition of the great arteries; *US*, ultrasound; *VACTERL*, vertebral, anorectal, cardiac, tracheoesophageal, renal, and limb anomalies; *VM*, ventriculomegaly; *VSD*, ventricular septal defect.

*Shelmerdine et al. Postmortem microfocus computed tomography for noninvasive autopsies. Am J Obstet Gynecol 2021*.
